# Supplementary material for: Are carbon emissions trading and green financial instruments synergistic? -Comprehensive quantitative research based on content analysis
Source: PLoS One. 2024 Mar 7;19(3):e0298601. doi: 10.1371/journal.pone.0298601 (PMC10919601; doi:10.1371/journal.pone.0298601)
Supplement: S1 Data — (ZIP) [file pone.0298601.s001.zip › Data/地级市Z_F工作报告.zip!/╜¡╦╒/╨∞╓▌╩╨/└┤╫╘╣1⁄2╓┌║┼í╛┬φ┐╦╩2╛▌═°í┐_2013.docx]

各位代表：

现在，我代表市人民政府向大会作工作报告，请予审议，并请市政协各位委员和其他列席人员提出意见。

一、2012年工作回顾

过去的一年，面对复杂严峻的国内外形势，全市上下在省委、省政府和市委的正确领导下，紧紧围绕“两快两带三先”的新要求，始终坚持稳中求进、好中求快的工作导向，牢牢把握主题主线，深入实施“八项工程”，全力推进“三重一大”，沉着应对挑战，积极抢抓机遇，有力推动了经济社会平稳较快发展。

（一）争先进位成效明显，经济综合实力稳步壮大

经济规模跃上新台阶。全市主要经济指标（预计，下同）增长继续高于全省平均水平，部分指标增幅全省领先。地区生产总值有望突破4000亿元、增长13%左右。公共财政预算收入实现366.8亿元、增长15.2%，其中税收收入增长19.8%，税占比较上年提高3个百分点。固定资产投资完成2685亿元、增长22%，其中工业投资1477亿元、增长21.5%，宿新高速、徐工大吨位起重机、卡特彼勒大挖掘机等重大项目如期竣工，亿吨大港、必康新医药等重大项目顺利推进。城乡消费同步拓展，社会消费品零售总额达1320亿元、增长18%左右。县域经济和民营经济快速发展，五县（市）地区生产总值和公共财政预算收入占全市比重分别达46%、44.8%；全市民营经济实现增加值2300亿元、增长14%。

产业转型升级深入推进。规模以上工业增加值突破2000亿元、增长17%，六大千亿元工业产业产值超8000亿元。服务业增加值达1650亿元、增长13.5%，现代服务业集聚区新增入驻企业503家。粮食产量实现“九连增”，荣获“全国粮食生产先进市”称号。高效设施农业新增面积、总面积保持全省第一，新增省级农产品加工集中区4个，黄河故道综合开发启动实施。高新技术和新兴产业产值分别完成3600亿元和3300亿元、均增长80%；全社会研发投入增长20%，规模以上工业企业研发机构覆盖率达85%。全市引进高层次人才2700多人、其中国家“千人计划”专家17人，首次入选省创新团队2家、实现了该项目的重要突破。新增2家百亿元工业企业、5家五十亿元服务业企业（市场），徐工集团营业收入超千亿。

改革开放迈出更大步伐。镇级行政管理体制改革试点有序实施，云龙湖风景区管理体制改革顺利完成。事业单位分类改革加快推进，精简市直事业机构61家。医药卫生体制改革进一步深化，以我市为中心的淮海经济区核心区8个城市实现新农合异地结算。财税金融体制改革不断加快，“营改增”试点顺利推进，农信社改制组建银行工作基本结束，村镇银行实现县域全覆盖。开放型经济加快发展，实际到账注册外资完成17亿美元、增长15%；进出口超千万美元的企业达120家、同比新增49家，全市自营出口达62亿美元、增长50%。

集聚发展能力进一步增强。各类开发园区实现业务总收入8676亿元、增长50%。徐州经济技术开发区成为工程机械全国知名品牌创建示范区，获批创建国家生态工业示范园区；徐州高新区成为苏北首家国家高新区；泉山经济开发区晋升省级开发区，沛县绿色食品产业园、睢宁白色家电产业园、新沂新型材料产业园获批省级特色园区，丰县在我省率先建成国家级出口果品质量安全示范区，邳州国家级大蒜出口基地创建通过商务部初审，沛县微山湖千岛湿地景区、吕梁山旅游度假区获批省级旅游度假区，新沂窑湾古镇成功创建国家4A级景区。矿大科技园成为全省唯一一家国家A类大学科技园，徐州医学院生物技术和新医药产业园获批省级科技产业园，大学生创业园获首批国家大学生创业示范基地称号；徐州软件园、徐州工业园科技创业园获批国家级、省级科技企业孵化器。

（二）城乡面貌大幅提升，一体化建设扎实开展

中心城市功能更加完善。启动新一轮城市总体规划修编，制定“八大中心”建设意见和专项规划，区域中心城市建设的目标定位和实现路径进一步明晰。243项城建重点工程进展顺利，项目总量和投资额均创历年新高，徐贾快速通道、彭城欢乐世界一期等重大项目投入使用，珠山景区、植物园、潘安湖湿地公园等精品园林景观建成开放，三环东路高架、韩山隧道、微山湖快速通道等道路工程加速推进，苏宁商务广场、中央国际广场、八里家居中心等商贸项目稳步实施，城市轨道交通等重大前期项目取得突破性进展，观音机场开通至泰国曼谷的国际航线。网格化城市管理体系逐步健全，市容专项整治效果明显，中山北路、民主路成为省级示范路，“数字化城管”通过省级验收并被国家住建部列为科技示范项目，我市荣获“江苏省城市管理优秀市”称号。

城乡建设协调推进。各县（市）城乡规划体系不断完善，组织实施了一大批基础设施和功能性项目，城乡面貌明显改观。投入资金23.2亿元重点加强30个中心镇建设，完成基础设施、社会事业、生产服务三大类项目87个，第一批10个中心镇达到验收标准。投入资金35.6亿元新创建新农村示范村50个，25个村被评为三星级康居村，8个村被评为省村庄建设和环境整治示范村。镇区环境“五整治、五提升”初见成效，村庄环境整治年度任务全面完成，京沪高铁沿线环境综合整治扎实开展。完成农村水利投资9亿元，解决了44万农村人口饮水安全问题，改造卫生厕所15万座。

生态建设进一步加强。积极开展PM2.5监测试点，大力实施污染防治设施提标改造，完成7450辆高排放机动车淘汰任务，市区环境空气质量良好率达90%。南水北调一期工程完成序时进度，截污导流工程投入运营，市区污水集中处理率达87%以上，重点断面水质稳定达标，功能区三类以上水体占比达76%。单位GDP降耗和主要污染物总量减排完成年度任务。绿化荒山2.2万亩，新增造林12万亩，改造绿地1500公顷，建成潘安湖等6个省级湿地公园，10个镇和11个村通过国家生态镇（村）考核验收，荣获“国家森林城市”称号，国家生态市创建扎实推进。我市被列入全国工矿废弃地复垦利用试点城市。

（三）民生民计持续改善，社会建设得到加强

人民生活水平不断提高。城乡居民收入分别达22100元和10900元，均增长12.3%。启动新一轮扶贫开发，实现农村低收入人口建档立卡全覆盖，发放扶贫小额贷款10.2亿元；对359个经济薄弱村全部制定了帮扶规划，一批帮扶项目相继实施。新增城镇就业12万人、失业人员再就业4.7万人、农村劳动力转移6万人，登记失业率控制在2.46%，大学生创业企业发展到2740家。“全民社保”框架体系基本形成，城乡居民养老保险参保及养老金发放实现动态全覆盖，新农合政策范围内报销比例达79.3%、位居全省第一，城乡低保标准分别提高到每人每月440元和270元。开工建设保障性住房10317套，竣工5810套，发放廉租住房租赁补贴2336户，建成棚户区改造安置房6707套，新建农村偏远学校教师公租房1056套。年初确定的三大类70件实事完成年度任务，投放7500辆公共自行车等实事受到群众普遍欢迎。

社会事业健康协调发展。教育优质均衡发展稳步推进，7个县（市）区创建国家义务教育基本均衡县（市、区）工作通过省级评估，新建改建农村中小学119所、幼儿园112所，超额完成校舍安全工程三年计划任务，农村中小学生上下学交通安全保障水平明显提高；生物工程职业技术学院成功升格，我市被确定为第二批省职业教育创新发展实验区。徐医附院新病房楼建成使用，中心医院新城区分院、第一人民医院和市精神病院迁建等项目有序推进，30家社区卫生服务中心完成升级改造，标准化卫生室基本实现行政村全覆盖。精心打造“舞动汉风”品牌，文化产业发展明显加快，一批文艺精品在国家和省获奖，社区文化共享工程基层服务点实现城区全覆盖，公共文化设施基本做到免费开放，我市被评为全国文化体制改革先进地区。行政村健身设施提档升级工程全面完成，奥体中心建设加快推进，第十八届省运会备战训练和筹办工作稳步开展，成功举办第一届市全民健身运动会和第二十届市运会。村（社区）综合服务中心建设任务圆满完成，服务功能进一步提升。人口计生综合改革深入推进，计划外生育反弹和出生人口性别比偏高的势头得到初步遏制，出生强度持续回落。再次荣膺“全国双拥模范城”称号，顺利实现“七连冠”目标。残疾人“强基育人”工作成效显著，镇村两级残疾人组织建设全国领先。妇女儿童、青少年、关心下一代、老龄等事业持续发展，人防、民兵、民族宗教、档案、外事、侨务、台港澳事务、统计、审计、机关事务、广播电视、淮塔管理、住房公积金管理、新闻出版、气象、援疆等各项工作都取得了新成绩。

社会管理创新得到强化。深入开展“三解三促”、“大接访”和“百日百案”活动，扎实推进“四项排查”，稳定风险评估数量和评估质效均居全省前列，非法集资专项治理等社会管理创新项目实现重点突破，来市上访总量和进京上访登记量不断下降。社会治安形势持续改善，严重刑事案件数量降至10年来最低。积极开展“幸福家园”创建，建成居家养老服务中心1300个，市养老信息服务中心投入使用，65%的城市社区和40%的农村社区达到和谐社区标准，5个社区被命名为国家级和谐社区示范单位。安全生产形势总体平稳，事故总量和死亡人数分别下降10%和11.2%。食品药品监管力度进一步加大，全市餐饮单位量化分级管理率达95%，药品生产经营企业监督检查覆盖率达100%。

（四）政府自身建设全面加强，行政效能明显提高

百姓办事“零障碍”工程扎实开展。设立400个协办中心和2100个协办岗位，建立了网络、电话、手机“零障碍”工程投诉平台，对群众申办事项实行全流程电子化监控和群众满意度即时测评，群众反映突出的29项办事障碍得到及时清除。112家市直单位修订完善相关制度160个，对85个事项的服务流程进行了优化，促进政务服务提速的长效机制不断完善。

“三位一体”政务服务新格局基本形成。大力推进审批服务提速，压缩行政审批事项113个，取消、下放审批事项20个，进驻行政服务中心事项审批时限压缩到5个工作日以内。着力推进便民服务提质，精心实施“市民卡工程”，主动拓展延时、预约、上门等便民服务。“12345”政府服务热线平台实现市县两级全覆盖，帮助解决群众诉求事项4万多件，综合服务功能全面加强。

法治廉洁政府建设再上新水平。自觉接受市人大及其常委会的法律监督和工作监督，主动接受市政协及社会各界的民主监督，深入推进依法行政，进一步细化了工作要求和标准，全市行政诉讼案件总量明显下降。开通全国首家政务公开和政务服务系统，12家试点单位的部门预算向社会公开。不断加强廉政主题教育，扎实推进“两个习惯”作风建设，积极开展领导干部“晒权力”试点活动，建立了集绩效、教育、监督、预防、惩戒于一体的勤廉评价系统，有力维护了为民务实清廉的政府形象。

各位代表，过去一年各项成绩的取得来之不易，这是全市人民同心同德、攻坚克难、开拓奋进的结果。在此，我代表市人民政府，向全市各族人民群众、向人民解放军驻徐部队、武警官兵和公安民警，向各民主党派、工商联、无党派人士和人民团体，向支持徐州现代化建设的香港特别行政区同胞、澳门特别行政区同胞、台湾同胞、海外侨胞和各界人士，向投身徐州经济社会发展的国内外投资者和企业家，表示衷心的感谢！

在肯定成绩的同时，我们也清醒地认识到，当前我市经济社会发展不充分、不平衡、不可持续的问题还比较突出，尤其是在经济下行压力加大的严峻形势下，部分企业经营困难明显加大，财政增收节支压力明显加大，居民收入持续增长压力明显加大，一些经济指标增速同比明显趋缓。公共服务供给与民生改善需求之间的差距仍然较大，教育、就业、医疗、住房、社会保障等事关群众切身利益的问题还不少。城市管理水平和城乡文明程度有待进一步提高，交通拥堵问题亟待解决。城乡之间、区域之间的发展尚不平衡，经济发展与资源环境约束加剧的矛盾比较突出。一些公务人员的服务意识不强，解决问题的能力和办事效率不高。在今后的工作中，面对推进跨越发展、科学发展、和谐发展的多重艰巨任务，如何更加有效地统筹解决好上述问题，是我们面临的重大考验。我们必须进一步凝心聚力谋发展、强化创新抓落实，以更强的执行能力、更高的工作标准和更优的服务效能，努力推动全市经济社会发展再上新水平！

二、今年政府工作的主要任务

今年是深入贯彻落实党的十八大精神的开局之年，是实施“十二五”规划承前启后的关键一年，也是在更高水平上全面建成小康社会的重要一年。综观宏观形势，我们仍处于可以大有作为的重要战略机遇期，具备许多有利条件和积极因素，但面临的困难和挑战依然较多。我们要看到，当前世界经济低速增长的态势仍在延续，在经济国际化不断加快的大背景下，产业转型升级的任务更加繁重，保持经济平稳较快发展压力较大。我们更要看到，国家继续实施积极的财政政策和稳健的货币政策，宏观调控将不断加强和改善，特别是党的十八大为推动科学发展进一步指明了方向，提供了强有力的支撑和保障。近年来，我市坚定不移地推进“三重一大”建设，积蓄了强大的发展后劲。只要我们切实增强紧迫感和忧患意识，始终坚定信心、抢抓机遇、攻坚克难，一定能够夺取新一轮发展的主动权。

做好今年的政府工作，要全面深入贯彻落实党的十八大精神，高举中国特色社会主义伟大旗帜，坚持以邓小平理论、“三个代表”重要思想和科学发展观为指导，按照“五位一体”的总布局，紧紧围绕“两快两带三先”的战略部署，始终突出主题主线，以提高经济增长质量和效益为中心，稳中求进、开拓创新、扎实开局，坚持又好又快工作导向，深入实施“八项工程”，集中力量抓好“三重一大”，坚定不移深化改革开放，全力以赴稳增长、转方式、调结构、抓创新、惠民生、促和谐，持续推动全市经济社会总量指标进位、速度指标争先、结构指标提升，在更高层次上振兴徐州老工业基地，在全面建成小康社会新征程上迈出更加坚实的步伐。

今年经济社会发展的主要预期目标是：地区生产总值增长13%，公共财政预算收入增长15%，固定资产投资增长22%，社会消费品零售总额增长16%，实际到账注册外资和进出口总额均增长15%左右，城市居民人均可支配收入和农民人均纯收入均增长12%以上，居民消费价格涨幅不高于全省平均水平，新增就业人口10万人，城镇登记失业率控制在4%以内，节能减排完成省定任务，人口自然增长率控制在6％以内。

为顺利实现上述目标，我们将着力抓好以下几个方面的工作：

（一）一心一意惠民生。改善民生既是政府工作的方向，也是人民群众自身奋斗的目标。办民生实事既要尽力而为，又要量力而行，把有限的财政资金更多地用到改善民生上，同时引导广大群众树立劳动致富改善生活的理念，共同创造幸福美好的生活。要深入实施民生幸福工程，立足当前办好三大类100件民生实事，着眼长远构建“六大体系”。

在增加居民收入上下功夫。突出高校毕业生、农民、企业职工、中低收入者和困难家庭等重点群体，进一步拓宽就业增收路径，多渠道增加财产性收入，扎实推进居民收入六年倍增计划。健全完善政策扶持、就业培训和创业服务机制，开发更多的就业岗位，强化就业和劳动技能培训，抓好15分钟公共就业服务圈等基层平台建设，推动“充分转移就业镇村”创建，年内新增农村劳动力转移5万人。引导劳动者转变就业观念，以促进创业带动就业，抓好大学生创业孵化基地和产业化基地建设，努力实现更高质量、更加充分的就业。推进新一轮扶贫开发，切实增强经济薄弱村自我发展能力，不断壮大集体经济实力，年内力争26.8万农村低收入人口实现脱贫，108个经济薄弱村实现新“八有”目标。

在强化社会保障上下功夫。把人人享有基本社会保障作为优先目标，继续扩大城乡社会保障覆盖面，确保主要险种覆盖率保持在95%以上，城镇居民医保政策范围内住院支付比例、新农合住院政策补偿比例巩固提升，城乡低保标准保持苏北领先。完善市场配置和政府保障相结合的住房制度，加强保障性住房建设和管理，新建第六期17.3万平方米经济适用房，引导社会力量参与公租房、廉租房建设。完善社会救助体系，大力发展慈善事业，按照不低于城乡居民收入增幅提高“三无”人员和孤儿供养标准。深入推进老龄事业发展，完善社会养老服务体系，加快老年公寓、居家养老服务中心、农村老年关爱之家、城市社区小型托老所等服务设施建设，为主城区低保和特困家庭60周岁以上老人购买公益性保险。积极落实惠民殡葬制度，抓好平价公墓建设。

在完善公共服务上下功夫。坚持教育优先发展，合理配置教育资源，提高教师队伍的业务能力和师德水平，大力提升各级各类教育办学质量。优化中小学布局，继续实施中小学校舍安全工程和农村偏远学校教师公租房工程，新建改建中小学44所、幼儿园118所，创建省市级优质幼儿园80所。加快区域教育基本现代化建设，创建110所义务教育现代化学校，推进普通高中优质特色发展，确保高中阶段毛入学率达95%以上，所有县（市、区）通过国家义务教育基本均衡县(市、区)评估验收。支持特殊教育发展，加快职业教育创新发展实验区建设，创建省首批高水平现代化职业学校3所、示范性实训基地10个。深化公立医院改革，鼓励社会办医，创建10个以上省级示范乡镇卫生院，继续推进社区卫生服务中心提档升级，加快市第一人民医院迁建和新里程国际医院、徐医附院开发区分院建设，打造区域性医疗中心。大力发展文化事业，完善公共文化服务体系，加快文化强市建设。深入开展全国文明城市创建，持续提高城乡居民文明素质。抓好第十八届省运会筹办工作，建成奥体中心；实施体育惠民“百千万工程”，推进城市社区“10分钟体育健身圈”和全民健身中心建设，争创体育强市。深入开展放心消费先进示范景区、放心粮油店、便民餐饮示范店（街）、放心药店、价格诚信店等创建活动，全力打造安全放心的消费环境。抓好残疾人托养机构建设，开展帮扶残疾人系列活动。坚持计划生育基本国策，提高出生人口素质，优化出生人口性别比。推进未成年人、农民等重点人群科学素质行动，不断提升全民科学素养。巩固双拥创建成果，进一步密切军政军民关系。统筹抓好其他各项事业，为群众提供更加完善的社会服务。

（二）千方百计稳增长。把稳增长作为经济工作的突出任务，把扩大内需作为战略基点，进一步拓展外需市场，积极破解各类瓶颈制约，促进经济平稳较快发展。

加快推进重大项目建设。年内计划实施重大项目220项、总投资4043亿元，其中当年实施200项、年度投资972.8亿元。抓住国家加快新型城镇化的机遇，重点推进郑徐客运专线、三环东路北路西路高架、城市轨道交通、亿吨大港、“两河两路”、黄河故道综合开发、五环路等26项重大基础设施项目。着眼促进经济稳定增长和产业结构优化，重点推进温氏畜牧农业示范基地、徐工重型汽车、徐烟异地改造、必康新医药、龙英纺织、万科城等164项重大产业项目。积极推进徐菏铁路、观音机场二期扩建等20项前期项目。有效破解用地、资金、环境等瓶颈制约，全力保障项目顺利实施。

积极推动各类园区集聚集约发展。进一步提升开发园区的承载能力、产业层次、产出比重和项目集聚程度，强化争先进位，更好地发挥引领示范作用。重点支持徐州经济技术开发区建设国家级一流开发区和国家级生态工业园，壮大以高端工程机械为主的装备制造、以光伏光电为主的新能源、以高端生产性服务业为主的现代服务业三大千亿级产业集群，加快发展生物医药、新材料等五大新兴产业。高水平推进徐州高新区“六大园区”建设，加大政策支持力度，积极打造区域性创新中心和科技成果转化基地，争创省级科技金融合作示范区和国家级生态工业园。高标准制定泉山经济开发区发展规划，集中发展现代物流、都市型工业、休闲旅游业等主导产业，加快培育市区西部重要经济增长极。合力推进其他各省级经济开发区、旅游度假区和南北共建园区发展，大力培育特色产业，加快形成功能互补、产业互动的发展格局。

大力强化招商引资和市场开拓。牢牢把握发展实体经济这一坚实基础，在强化支持本土企业的同时，坚持“四招四引”，加强产业链招商、专题招商和以商引商，精心组织第十六届投资洽谈会暨第七届汉文化旅游节等重点招商活动，抓好项目推介和签约项目的跟踪落实。加快建设专业素质强、人员相对稳定的招商团队，着力提高招商效益，争取各县（市）都有一批10亿元或1亿美元以上的产业项目落地。加大对进出口企业的帮扶力度，推动徐州工程机械申报国家级出口基地，鼓励有条件的企业争创国家级出口品牌，积极培育国际化大型企业集团。

（三）坚定不移调结构。坚持做大产业规模与优化产业结构互动并进，大力实施产业转型升级工程，不断延伸产业链条，切实提升产业发展的层次、质量和效益。

加快新型工业化步伐。以规模、品牌等“六个提升”为抓手，壮大六大工业千亿产业的综合实力和市场竞争力，力争规模工业总产值过万亿。加速培育战略性新兴产业，保持高新技术产业产值增幅全省领先，力争年内高新技术产业、新兴产业产值分别达4300亿元和4000亿元。坚持扶大扶优扶强，支持徐工集团强化资本运作、实施对外并购和产业基地建设，加快推动中能协鑫开展技术升级和新项目建设，鼓励优势企业开展合资合作、加速规模扩张，强化传统企业技术改造。进一步提高铜山工程机械、钢铁冶金及贾汪建材等产业集聚度，支持贾汪创建国家资源枯竭城市转型发展示范区。推动信息化和工业化深度融合，推进信息技术在企业研发、生产、销售和管理等环节的应用，争创国家级、省级“两化融合示范基地”。

狠抓现代服务业发展。精心打造中心商圈、高铁生态商务区、新城区总部经济区等现代服务业活力板块，加快推进万达广场、鼓楼广场、淮海西路商业街等重大服务业项目，继续实施制造业企业主辅分离，促进商贸、物流、金融等服务业扩容提质，推动软件、云计算、物联网等新兴服务业加速扩张。强化旅游产业功能配套和景区景点建设，推动云龙湖风景区、邳州艾山九龙风景区创建国家5A级景区，启动“智慧旅游”项目与旅游咨询中心建设。完善优进劣出的动态调整机制，推进现代服务业集聚区提档升级，构筑一批各具特色的服务业发展载体。坚持一手抓文化事业繁荣，一手抓文化产业发展，深入实施“舞动汉风”工程，精心组织文艺精品创作，重点发展演艺娱乐、文艺服务等文化业态，加快老徐州历史文化片区、影视动漫基地、龟山博物馆景区等载体建设，争创国家和省级文化产业示范园区。

大力提升农业现代化水平。深化农村改革，增强农村经济发展活力。加快高标准农田和农村水利基础设施建设，实施150万亩高亢山区水源工程，大力推广农业机械化，增强农业综合生产能力。抓好万顷良田建设，继续实施高效设施农业“2020”工程，促进现代农业规模化发展。加强铜山、沛县国家级现代农业示范区和7个省级现代农业产业园建设，大力扶持农业龙头企业，鼓励农民专业合作组织发展，培育新型经营主体，有序推进土地流转，积极开展农田托管，发展多种形式的适度规模经营，不断提升农业集约化、专业化、组织化和社会化水平。突出农业科技创新，加强农产品质量安全监管，强化无公害农产品和绿色食品基地建设，做好农产品质量和名牌认证，完善农产品销售网络。深入实施黄河故道综合开发，坚持水利、交通、农业、生态、文化旅游、扶贫“六位一体”，系统推进中泓贯通、土地整治等八大工程，打造现代农业示范带。

加速壮大县域经济。完善县域经济发展规划，加强对土地利用总体规划修编、产业布局和发展规划编制的指导，进一步明晰产业发展重点和城市发展定位。鼓励沛县、新沂、邳州提速发展，深化“突破睢宁”战略内涵，加快推动“丰县崛起”，强力促进县域发展争先进位，加快建成实实在在、不含水分、老百姓认可的小康社会。支持丰县盐化工和电动车、沛县煤化工和铝加工、邳州板材和再生资源、睢宁皮革皮具和白色家电、新沂新医药和精细化工等特色产业提质增效，做强县域经济产业支撑。

（四）全力以赴抓创新。把创新作为改革发展的着力点和突破口，强化技术创新和体制机制创新，深化重点领域和关键环节的改革，突出解决制约经济持续健康发展的重大结构性问题。

大力推动科技创新。突出企业技术创新主体地位，提高原始创新、集成创新、引进消化吸收再创新能力，加强商业模式创新，坚持开放式创新，更加注重协同创新。抓好传统优势企业技术改造和产品创新，新增省级高新技术企业40家以上。实施自主研发重大项目20项，组织研发省级高新技术产品350项以上，力争专利授权量突破一万件，启动国家知识产权示范市创建工作。开展博士后和海外留学人员科技项目洽谈、“百所名校徐州行”等产学研对接活动，支持企业牵头实施重大科技项目、承建国家和省级科技创新平台、到海外设立研发机构，年内新建校企联盟200家，新增省级以上研发机构30家。推动矿大科技园加快发展，促进师大科技园创建国家级大学科技园，建设好徐州医学院、徐州工程学院、江苏工业职业技术学院等省、市级大学科技园。完善人才政策体系，用好本地人才，加速引进高层次创新创业人才和紧缺人才，加强知识产权保护，努力打造区域性人才高地。

大力推动国有企业体制创新。坚持增量式改革及淘汰落后产能相结合，积极引进战略合作伙伴，稳妥推进国有企业改制和经营机制创新，加大国有企业优化组合力度，积极推行年薪制改革，激发企业内在发展活力。加快徐工集团改制步伐，着力整合化工、轻工、机电建材、建筑资产四家资产经营公司，继续做好物资集团债务处置工作，认真解决改制企业遗留问题。加快建设新的区域生产资料市场，培育壮大苏山木材市场。进一步完善国有资产管理体制，做大做强资产规模，促进国有资产保值增值，做好国有资产收益收缴工作。

大力推动投融资体制创新。强化政府投融资平台建设，有效整合各类资源，着力提高投融资运作水平，确保市级投融资平台融资规模达到400亿元。引导金融机构创新金融产品，扩大直接融资渠道，深化金融顾问制度，健全金融监管协作机制，改善区域金融生态环境，有效降低企业特别是中小企业的融资成本，确保贷款增速高于全省平均水平、新增非信贷融资额不少于新增贷款额。加快金融集聚区建设，引进更多的金融机构落户徐州，推动省级金融机构在徐州设立信贷审批分中心，支持各类资本创办创业投资、风险投资企业。

大力推动行政管理体制创新。稳妥推进市区行政管理体制改革，进一步简政放权。深化经济发达镇行政管理体制改革试点，开展赋予重点中心镇县级管理权限试点，激发基层发展活力。加快事业单位改革，基本完成分类改革任务。切实处理好政府和市场的关系，保障各种所有制经济依法平等使用生产要素、公平参与市场竞争、同等受到法律保护，强化对民营企业和成长型中小企业的政策扶持，大力消除有形和无形壁垒，放宽市场准入，完善市场环境。

（五）统筹协调建城乡。以整体增强区域性中心城市的集聚辐射功能为目标，着力在城乡规划、基础设施、功能配套和公共服务方面实现统筹推进、有序发展。

持续提升中心城市现代化水平。围绕建设区域性“八大中心”，加快推进三环东路北路西路高架、新淮海西路等道路畅通工程，启动建设徐沛快速通道、徐明高速，开通至韩国航线，确保丰沛铁路竣工通车；启用徐州高铁综合客运枢纽，抓好北区长途客运站等功能性项目建设。实施市区林荫路、道路绿化提升工程以及公园服务半径达标工程，开展泉山森林公园等敞园改造，新建大黄山森林公园、三八河带状公园。加快新城区沿昆仑大道、汉源大道和环大龙湖“三条轴线”开发，全面推进高铁生态商务区二期建设，加速铜山、贾汪融入主城区步伐，推动城市组团发展。着力强化城市管理，编制“智慧城市”规划，完善数字化城管监控系统，加强违法建设制止和管理，有序实施市容市貌综合治理，做好居民小区及背街小巷的专项整治，深入开展“外创示范路、内创幸福家园”活动。抓好城乡环卫基础设施建设，完善垃圾收运体系。突出公交优先，大力治理城市非法营运出租车，新增停车泊位9000个、公共自行车7500辆，新建改造一批公交港湾，努力缓解市区交通拥堵状况。继续推动淮海经济区核心区合作发展，加速一体化进程。

着力推进新型城镇体系建设。按照集约、智能、绿色、低碳的新型城镇化要求，注重城镇化发展与产业布局有机衔接，抓好徐州特大城市和各县（市）总体规划修编，加快编制城镇控制性详规及专项规划，促进大中小城市和小城镇功能互补、集约发展。有序推进各县（市）老城改造和新区建设，大力实施“1+3个100万”工程，促进各县（市）每年出让1000亩建设用地，每年征收100万平方米土地、建设100万平方米安置房、销售100万平方米商品房，加快建成宜居宜业的中等城市。继续开展中心镇创建，完善乡镇分类考核机制，打造一批工业强镇、农业强镇、商贸旅游名镇。鼓励和引导农村居民向城镇转移，有序推进农业转移人口市民化。

扎实抓好新农村建设。继续推进新农村示范村创建，建成50个市级新农村示范村、25个三星级“康居乡村”。以“六整治、六提升”为抓手，完成2100个村庄环境整治任务。推动公共资源向农村延伸，建设农村公路200公里、农村桥梁100座，力争镇村公交覆盖率达到25%以上；疏浚县乡河道281条，清理村庄河塘土方1700万方；抓好农民饮水健康工程；实施农村改厕12万户、农村有线电视入户10万户。

(六)齐心协力促和谐。更加注重人与自然和谐共生、人与人和谐相处，加大生态文明建设力度，加强和创新社会管理，努力建设美丽徐州、和谐徐州。

更大力度抓好生态文明建设。全面推进资源利用方式转变，严格耕地保护，完善土地资源空间开发保护制度，降低能源、水、土地的消耗强度，提高农作物秸秆等生物资源综合利用水平。严格落实节能减排措施，坚决关停规模以下“两高”企业，实施一批节能改造示范工程，确保年耗能3000吨标准煤以上企业节能改造达到35%以上。加快新沂、丰县、沛县、睢宁的水资源化利用工程建设，强化重点断面水质保护区管理，实施荆马河、老房亭河、顺堤河、丁万河等一批河湖水环境综合整治，大力提升污水处理厂建设和管理水平，改造完善三环北路及西路污水管网和市区18个片区排水线路，确保水质达到功能区标准。积极开展PM2.5监测，强化对机动车尾气排放、施工扬尘、工业废气烟尘的治理。坚持建管并重，大力推进国家生态市创建，绿化荒山1.9万亩，开展京沪高铁沿线绿化和26个采石宕口生态整治，加快吕梁山风景区、黄河故道绿色生态长廊、沛县千岛湿地建设，全面完成30个国家级生态镇创建任务，力争沛县、铜山区、贾汪区建成省级生态县（区）。加强对重金属企业污染的治理整顿，推进三环路以内化工企业和二环路以内规划搬迁工业企业退城入园，着力解决关系民生的突出环境问题。

更大力度加强和创新社会管理。深入实施社会管理创新“四治理、三提升”工程，加强基层社会管理和服务体系建设，继续抓好村（社区）服务中心建设，建立60个规范化县（市）区、镇（街道）社会管理综合服务中心，确保城乡和谐社区达标率分别达75%和55%，着力提升基层组织协调事务、化解矛盾的能力，充分发挥其功能和作用。健全重大决策社会稳定风险评估机制，完善信访工作制度和人民调解、行政调解、司法调解联动的工作体系，畅通和规范群众诉求表达、利益协调、权益保障渠道，严厉打击非法集资，加大企业改制、征地拆迁、涉法涉诉等重点问题解决力度。深化治安防控体系建设，加强农村中心警备室和城市社区警备室建设，大力预防和依法惩治违法犯罪。突出校园周边环境综合治理，进一步提高校园安全水平。强化食品药品生产经营源头管理和环节监管，开展药品药械打假治劣安全专项行动，严格食用油、食品添加剂、生鲜肉等重点品种的跟踪检查。加强安全生产基础和监管体系建设，着力抓好重点领域隐患排查治理，坚决遏制较大以上事故的发生。

三、加强政府自身建设

为人民服务是各级政府的根本宗旨，为经济社会发展服务是各级政府的首要职责。我们一定要确立长期艰苦奋斗的思想，既要开拓奋进，又不能急于求成，不盲目攀比指标数字，坚决反对弄虚作假，勤勉尽责，永不懈怠，努力提高推动发展、统筹协调的能力，努力建设人民更加信赖的政府。

（一）推动行政服务再上新水平。按照应进必进、精简高效的原则，再清理、减少、调整一批审批事项，进一步强化窗口的服务功能，加快推动行政审批项目全部纳入行政服务中心办理。建设全市统一规范的公共资源交易中心，提高资源配置质量和效率。强化便民服务中心建设，做好“市民卡”发放和管理工作；加大“12345”服务热线整合提升力度，积极吸纳直接服务民生的企业成为上线成员单位，打造“线报声屏网”立体联动服务体系，加快向社会服务特别是生活类社会服务领域拓展。认真做好我市第三次全国经济普查工作，为经济社会发展提供科学准确的统计信息支持和优质服务。

（二）坚持不懈改进工作作风。深入实施百姓办事“零障碍”工程，坚持思想教育、素质提升、铲除障碍、程序优化多措并举，构建更加方便快捷的长效服务机制。按照中共中央政治局和省委关于改进工作作风、密切联系群众的有关要求，制定贯彻落实办法，狠抓措施落实。坚持轻车简从、减少陪同、简化接待，深入基层察实情、解难题，推进“三解三促”活动常态化制度化，杜绝走过场和形式主义。切实改进文风会风，提倡少开会、开短会，少发文、发短文，严格控制以政府名义召开全市性会议和举行大型活动，加快电子政务建设，减少纸质文件，提高办文、办会、办事效率。深入开展创先争优活动，优化绩效考核评价体系，强化督查督办，加大行政问责力度，使“善操作、会落实、能创新、敢担当”成为政府工作人员特别是各级领导干部的鲜明特质。

（三）促进行政行为更加规范有序。把严格依法办事贯穿于政府工作的各个环节，重点加强行政执法规范化建设，严格落实行政裁量基准制度，认真清理基层执法项目，积极预防化解行政争议，不断提高依法行政水平。依法扩大政务公开，推行部门预算公开，做好政府信息发布工作，推进领导干部“晒权力”活动，增强政府工作的透明度和公信力。强化政府内部层级监督，更加自觉地接受市人大及其常委会的法律监督和工作监督、市政协的民主监督，高度重视社会公众监督和新闻舆论监督，切实加强改进行政监察和审计监督，确保各项工作在法律框架内开展。

（四）牢固树立清正廉洁的良好形象。厉行勤俭节约，严格“三公”经费管理，坚决制止铺张浪费，把有限的资源和财力用在推动发展急需的地方、用在改善民生最迫切的事情上。持续开展“两个习惯”主题教育活动，大力实施专项整治，坚决纠正损害群众利益的不正之风。坚持标本兼治、综合治理、惩防并举、注重预防的方针，认真落实党风廉政建设责任制，推进惩治和预防腐败体系建设，切实做到干部清正、政府清廉、政治清明。

各位代表，做好今年的各项工作，要求高、困难多、任务重，我们深感责任重大、使命光荣。让我们紧密团结在以习近平同志为总书记的党中央周围，全面贯彻落实省委、省政府和市委的决策部署，锐意进取，克难奋进，扎实工作，确保圆满完成全年各项目标任务，全力开创经济社会又好又快发展的崭新局面！
